# Acute post-orchiectomy pain does not reduce alpha rams’ interest in feed resources

**DOI:** 10.3389/fvets.2024.1299550

**Published:** 2024-03-19

**Authors:** Kauany Zorzenon Uzae, Pedro Henrique Esteves Trindade, Paula Zanin Rattes, Anna Laura de Sousa Campos, Leornado Garcia Bornal, Marina Belucci Teixeira, Henry David Mogollón García, Antônio Guilherme Pupulim, Renan Denadai, Eduardo dos Santos Rossi, John Patrick Kastelic, João Carlos Pinheiro Ferreira

**Affiliations:** ^1^Department of Veterinary Surgery and Animal Reproduction, School of Veterinary Medicine and Animal Science (FMVZ), São Paulo State University (Unesp), Botucatu, Brazil; ^2^Department of Population Health and Pathobiology, College of Veterinary Medicine, North Carolina State University (NCSU), Raleigh, NC, United States; ^3^Faculty of Veterinary Medicine, University of Calgary, Calgary, AB, Canada

**Keywords:** appetite, dominance, hierarchy, pain assessment, sheep

## Abstract

Sheep pain is an animal welfare issue monitored based on behavioral responses, including appetite. Dominant (alpha) males have priority for accessing limited feed resources, however, the effects of pain on feed interest in members of a group with defined social hierarchy are unknown. Our objective was to investigate effects of acute post-orchiectomy pain on alpha rams’ interest in accessing a limited feed resource. Eighteen rams were randomly housed in pens of 3 rams. After acclimation, the first 5-d (consecutive) battery of a behavior test was performed. In this test, 180 g of the regular diet concentrate was placed in a portable trough in the center of the pen; this feed was supplemental to the diet and represented a limited, albeit strongly preferable feed resource. Rams were filmed for 5 min after the feed introduction. Hierarchical levels (alpha, beta, and gamma) were defined based on the social hierarchical index according to higher initiator and lower receptor agonistic behaviors from the social network analyses. After 15 d, a second 5-d behavioral test battery was repeated. On the following day, alpha rams were castrated. Flunixin meglumine was given immediately before surgery and a final behavioral test was performed 8 h post-orchiectomy, concurrent with an expected peak in postoperative pain. For all recordings, the latency, frequency, and duration of time that each ram had its mouth inside the feed trough were recorded, and the Unesp-Botucatu sheep acute pain scale pain scale (USAPS) was applied. The social hierarchical index was highest in alpha rams, followed by beta and gamma. The pain scores were statistically equivalent across the 11 evaluation days for beta and gamma rams, whereas there was an increase in the final evaluation for alpha. There was no difference in latency, frequency, and duration between alpha, beta, and gamma rams across evaluations. We concluded that acute post-orchiectomy pain did not decrease alpha rams’ interest in accessing limited feed. Routine feeding offers a valuable chance to detect pain-related behavior using the USAPS in rams. However, dominance may confound appetite-related behaviors in assessing acute pain, as alpha rams’ interest in limited feed remained unaffected by the pain.

## 1 Introduction

The welfare of non-human animals has increasingly become a worldwide concern (1), with pain being well-known to reduce animal welfare and representing an important issue (2–4). Sheep are raised to supply the demand for meat and wool, and are commonly subjected to painful procedures, such as castration, tail docking, and mulesing (2, 5–7), as well as being widely used in experiments involving pain (2). In addition, sheep can suffer pain due to unintentional situations, such as dystocia, diseases, and injuries occasioned by poor handling or housing (8–10). In this context, an adequate pain diagnosis and treatment is essential (2, 7).

The International Association for the Study of Pain understands human pain as “an unpleasant sensory and emotional experience associated with, or similar to that associated with actual or potential tissue damage” (11). Thus, it is evident that pain is a multidimensional phenomenon involving sensitive, cognitive, and emotional components that can trigger feelings of animal suffering (12).

Although subjective phenomena are difficult to assess due to their multidimensional characteristics (7), painful sensations in animals can be assessed by whole-body behavior, facial expression and physiological paramenters changes (2, 7, 13). The unpleasant sensation caused by pain results in behavioral responses, such as lethargy, reluctance to move, and a lack of interest in feed (2). In particular, appetite is an easily visualized behavior in sheep production, as feed is offered daily at a specific time, and decreased appetite is a behavioral parameter for assessing the effects of pain in sheep (14–16).

Recently, a species-specific behavioral scale for pain assessment was validated for sheep (Unesp-Botucatu sheep acute pain scale; USAPS) using appetite after laparoscopy as one of the pain parameters (17). Although voluntary consumption of *ad libitum* feed by ruminants can be regulated by endogenous factors (e.g., pain), this control is also exerted by other mechanisms related to the characteristics of the diet and the environment in which it is offered (18). For example, social hierarchy can also influence the amount of feed ingested, with dominant males (alpha) having priority in accessing available resources (19). Therefore, dominance may be a confounding factor in pain assessment. However, to date, it appears that no studies have evaluated the interest in feed of alfa rams experiencing acute postoperative pain. This question has not yet been investigated even for alpha rams, which represent the hierarchical position most readily observed and distinguished from other positions. From a practical point of view, if dominance does not appear to represent a confounding factor in pain assessment, the moment of feeding could be an opportunity to detect appetite changes related to acute pain and improve the pain diagnosis on the farms.

We hypothesized that alpha rams, when experiencing acute postoperative pain, present decreased interest in the feed resource. Therefore, our objective was to investigate the influence of acute post-orchiectomy pain on the interest of alpha rams housed in trios to access limited feed resources.

## 2 Materials and methods

The study was approved by the host institution’s Ethics Committee on the Use of Animals (protocol: 0131/2021) and followed the Animal Research: Reporting of *In Vivo* Experiments guidelines (ARRIVE) (20). The rams and procedures presented below were not exclusive to the present study as they originated from a central animal reproduction study. In this way, we used the same rams and procedures, and only included additional behavioral tests to meet our objective. The use of the same group of animals for different studies contributes to the four Rs of animal experimentation (reduce, replace, refine, and respect) (Banks, 1995; Russell and Burch, 1959) and promotes animal welfare.

### 2.1 Rams and housing

Eighteen Santa Ines-Dorper clinically healthy crossbreed rams (*Ovis aries*), free of gastrointestinal parasites, from the same commercial farm and flock, 22.6 ± 2.6 mo of age and 36.7 ± 5.1 kg body weight, were used. The rams were randomly housed in groups of 3, in 6 pens (each 2 × 2 m), with a slatted wooden floor, *ad libitum* access to water, and fed a total mixed ration (60% concentrate and 40% Tifton 85 grass hay) offered twice daily, consistently at ~08:00 and 17:00. The diet was formulated to meet nutritional requirements for ram maintenance and growth.

### 2.2 Behavioral tests

**Figure 1:**
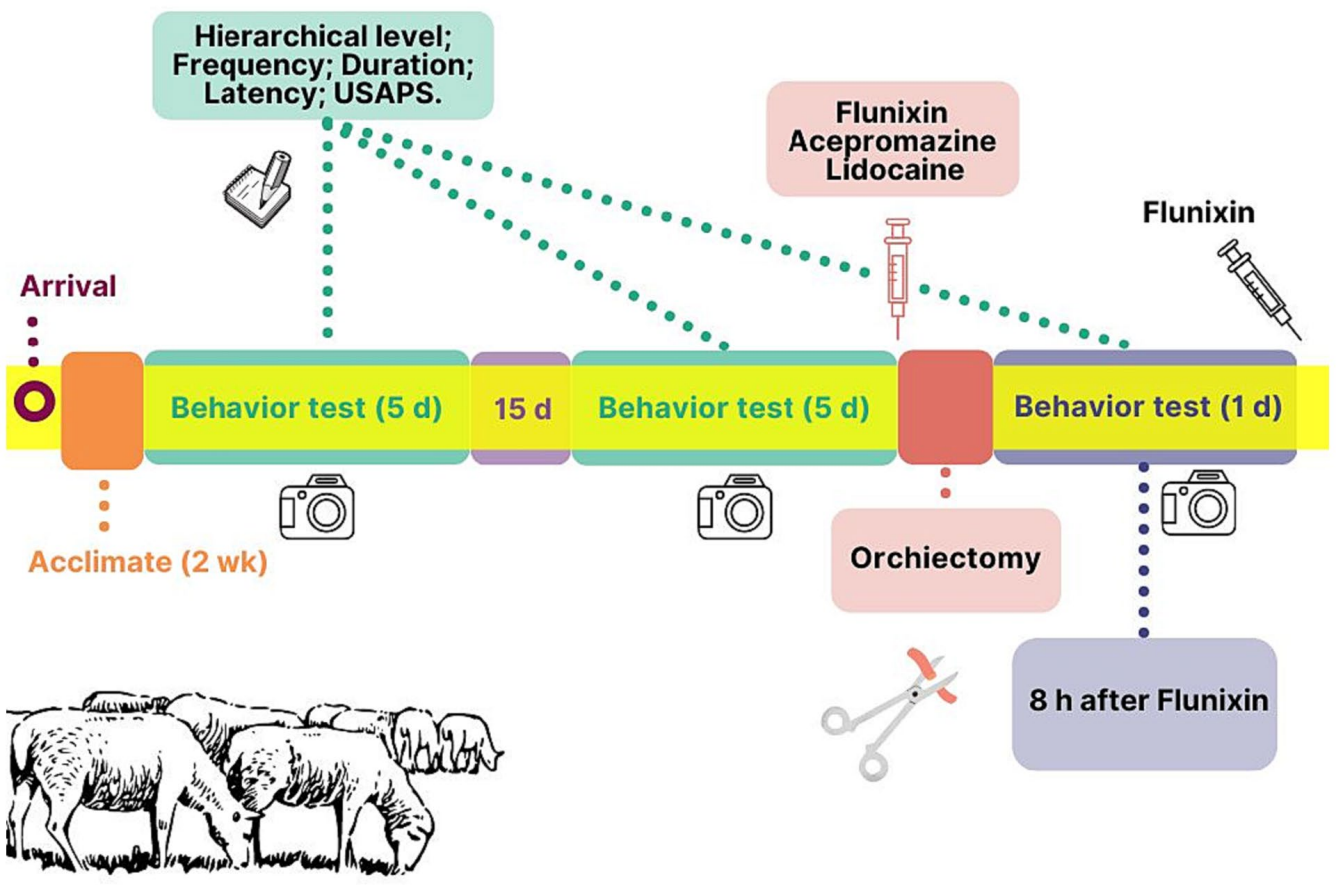
Study flowchart.

**Figure 2:**
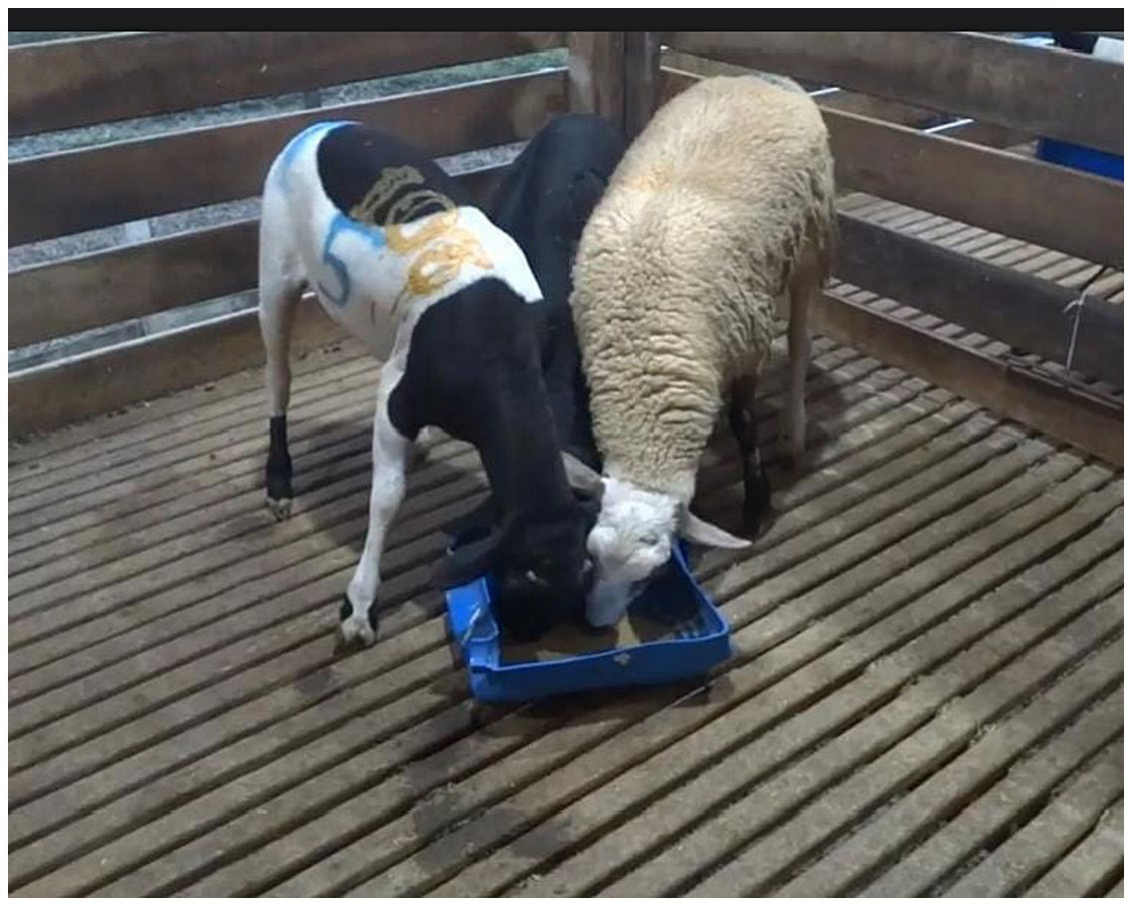
Illustration of the behavioral test in which a portion of concentrate is provided in an extra portable feed trough positioned in the center of the pen with three rams.

The study timeline is summarized in a flowchart (Figure 1). After 2 wks of acclimatization to the facilities, rams underwent two preoperative batteries of a behavioral test (1^st^–5^th^ and 6^th^–10^th^ assessments), interspersed by 15 d. Each battery consisted of 5 consecutive days of one 5-min test a day. The behavioral test was conducted at the same time (16:30) on each day. For the test, 180 g of the concentrate included as part of the regular diet was provided in an extra portable feed trough (37.6 × 29.1 × 25.7 cm) positioned in the center of the pen (Figure 2). This behavioral test was adapted from previous studies (21, 22). The behavioral test was filmed (Cyber-shot DSC-HX300, SONY Corporation, Brazil) for 5 min, starting from the placement of the feed trough on the floor. These procedures allowed us to establish each trio’s social hierarchical order, with alpha as the most dominant, beta as intermediate, and gamma as the least dominant.

The final behavioral test (11^th^ assessment) was performed 8 h post-orchiectomy of the alpha rams (16:30), at the expected peak of acute postoperative pain, based on the flunixin meglumine half-life (23). Immediately after the behavioral test, a second dose of flunixin meglumine was given to avoid the animals from suffering pain.

After the end of the experimental period, all rams were incorporated into the faculty flock.

### 2.3 Surgical procedures

On the morning following completion of the second battery of tests, only the alpha rams were castrated using a semi-open technique (24) (Figure 1). Preoperatively, 40,000 IU/kg of penicillin G benzathine and 2.2 mg/kg of flunixin meglumine were given intramuscularly, plus 0.2 mg/kg of acepromazine intravenously. After 15 min, 20 mL of lidocaine 2% with epinephrine were given, divided as follows: 5 mL in each spermatic cord and 10 mL in the scrotal sac incision site. Then, a pre-surgical scrub of the scrotal skin was performed, and the orchiectomy was consistently carried out by the same two veterinarians (AGP and ESR).

### 2.4 Video recording evaluation

Based on retrospective evaluations of the video, it was possible to collect the frequency of the following 5 social interactions, identifying the animal that initiated each behavior (initiator) from the one that received the behavior (receiver): (i) headbutts, when an animal (initiator) hits another animal (receiver) with its head vigorously, either by pushing with a neck movement or moving away at least 1 step from the receiver); (ii) stamping, when an animal (initiator) raises 1 of the front legs and immediately lowers it, hitting another animal (receiver), the trough or floor, being able to repeat the movement a few times in sequence, with each movement counted as one occurrence; (iii) fights, characterized by headbutting, front leg kick, and pushing while two animals faced each other, the initiator being the one who initiates the conflict and the receiver who receives it; (iv) mount or mount attempt, when an animal (initiator) raises the forelimbs and supports the thorax on the body of the other animal (receiver), characterizing one mount. If the receiver evades so that the initiator’s chest does not rest on its body, it characterizes a mount attempt; and (v) pushing, when one animal (initiator) exerts a forceful pushing movement against another animal (receiver), forcing it to move (19). The same evaluator (ALSC) collected all the social interactions observed. As detailed in the statistical methods, the above social interactions were used to determine the most dominant (alpha) ram from each pen.

The video recordings were also used to record the latent interval (seconds) between introduction of the feed trough and the moment that a ram touched it with its mouth, the frequency with which each ram put its mouth in the trough, and the duration (seconds) of the head into or above the trough area, either biting or chewing. In addition, the Unesp-Botucatu sheep acute pain scale (USAPS) (17) was applied. The USAPS comprises six behavioral items (interaction, locomotion, head position, activity, posture, and appetite) classified by three descriptive levels in each item (‘0’, ‘1’, and ‘2’). Level’ 0′ indicates normal behaviors (without association with pain), and levels’ 1′ and ‘2’ indicate behaviors proportional to pain intensity. In each assessment, the USAPS items were added to obtain the total sum indicative of pain intensity. A USAPS total sum greater than or equal to 4 indicates the need for analgesia (17). Tutorial videos of each USAPS behavior can be viewed at Animal Pain website^1^ or at the application Vetpain^2^ or in the previous publication (17). The same evaluator (KZU) collected the behaviors described above and the USAPS by randomly evaluating all videos, without knowing the moment observed (‘blind analysis’).

All behavioral measures described above were collected in all assessments (1st–11th) and for all alpha, beta, and gamma rams, regardless of whether or not they were castrated.

### 2.5 Statistical analyses

All statistical analyses were performed in R software with the RStudio integrated development environment [Version 4.0.2 (2020-06-22), RStudio, Inc.]. Functions and packages were presented in the “package::function” format based on the R programming language, and a significance of 5% was considered for all tests.

The frequency of initiator and receiver behaviors of the agonistic interactions (headbutts, stamping, fights, mounts or mount attempts, and pushing) recorded in the 10 preoperative sessions of all 18 rams were submitted to a social network analysis (SNA). From the number of initiations divided by the number of receptions of the SNA, a social hierarchical index was determined, whose increment indicated a higher frequency of initiation and a lower frequency of reception of social behaviors (sna::SNA). The alpha ram of each pen was determined by the highest social hierarchical index, while the gamma ram was the one with the lowest index and the beta the ram with the intermediate value. Then, to compare the social hierarchical index (outcome variable) between the hierarchical classifications (alpha vs. beta vs. gamma; explanatory variable), a multilevel linear model was conducted (lme4::lmer), including the pen as a random effect. The residual error of the model (stats::residuals) indicated adherence to a Gaussian distribution by quantile-quantile plot (stats::qqnorm), histogram (stats::hist), and by Shapiro–Wilk (stats::shapiro.test) and Cramer-von Miser tests (nortest::cvm.test) after Box-Cox transformation (lambda of −0.4244345) (car::powerTransform). Multiple comparisons were conducted in the *post hoc* test with Tukey’s test to compare the hierarchical classifications (lsmeans::lsmeans and multcompView::cld).

To explore multiple associations between variables that measured interest in the extra feed resource (latency, frequency, and duration) with pain assessment (USAPS), a principal component analysis (PCA) was conducted by a correlation matrix (stats:: princomp). Horn’s parallel analysis (psych::fa.parallel) was used to determine the optimal number of principal components (PC) to be retained in the PCA.

Modeling was conducted to investigate changes in variables over the three assessment periods (1st–5th vs. 6th–10th vs. 11th) and between the hierarchical classifications (alpha vs. beta vs. gamma). For this, a multilevel zero-inflated Poisson model (GLMMadaptive:: mixed_model) was used for USAPS, a multilevel linear model (lme4::lmer) was used for duration and latency, in which the residual error of the model indicated adherence to a Gaussian distribution by the methods described above, after Box-Cox transformation of latency (lambda of −0.3913293), and a multilevel generalized linear model adjusted by Poisson distribution was used for frequency. In all models, interactions of three periods of assessments and hierarchical classification were used as fixed effects, and the rams nested in the pen as a random effect. Multiple comparisons in the post-test of the model were performed as described above.

The analysis to estimate the sample size of alpha rams was conducted by setting 0.80 for power and 0.05 for significance level (stats::power.t.test). For the USAPS total sum at 8 h post-orchiectomy of the alpha rams, the delta (6.33) was reached by subtracting the mean calculated for the alpha rams (6.33) and beta rams (0.00) and the common standard deviation of all alpha and beta rams (3.11). According to the aforementioned limits, the sample size was estimated to be at least four rams per hierarchical classification (≅ 4.154542).

## 3 Results

**Figure 3:**
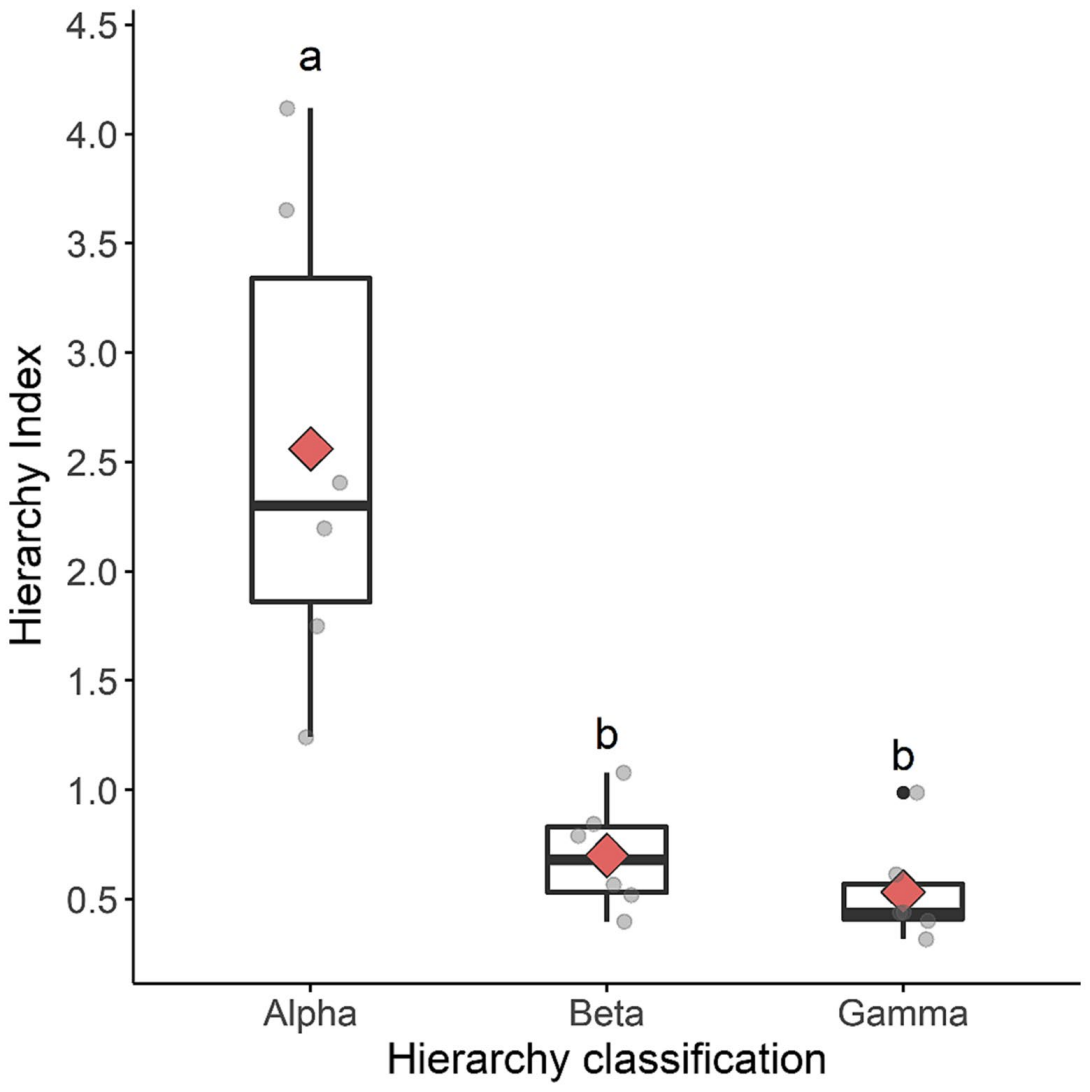
Box plot of differences in the hierarchical index among rams classified as alpha, beta, or gamma. The lower and upper bounds of the box represent the first and third quartiles of the data, respectively; the longer horizontal line inside the box indicates the median; the red diamond indicates the mean; gray circles indicate each individual ram; and lowercase letters indicate differences between classifications (a > b > c); multiple comparisons were conducted by Linear Models with post-test by Tukey’s test (*p* < 0.05) after Box-Cox transformation to meet the normality assumption; however, raw, untransformed data are presented.

The hierarchical index was higher in alpha rams and lower in gamma and beta rams, demonstrating that the hierarchical classification adopted in our study was able to identify the alpha ram (Figure 3).

**Figure 4:**
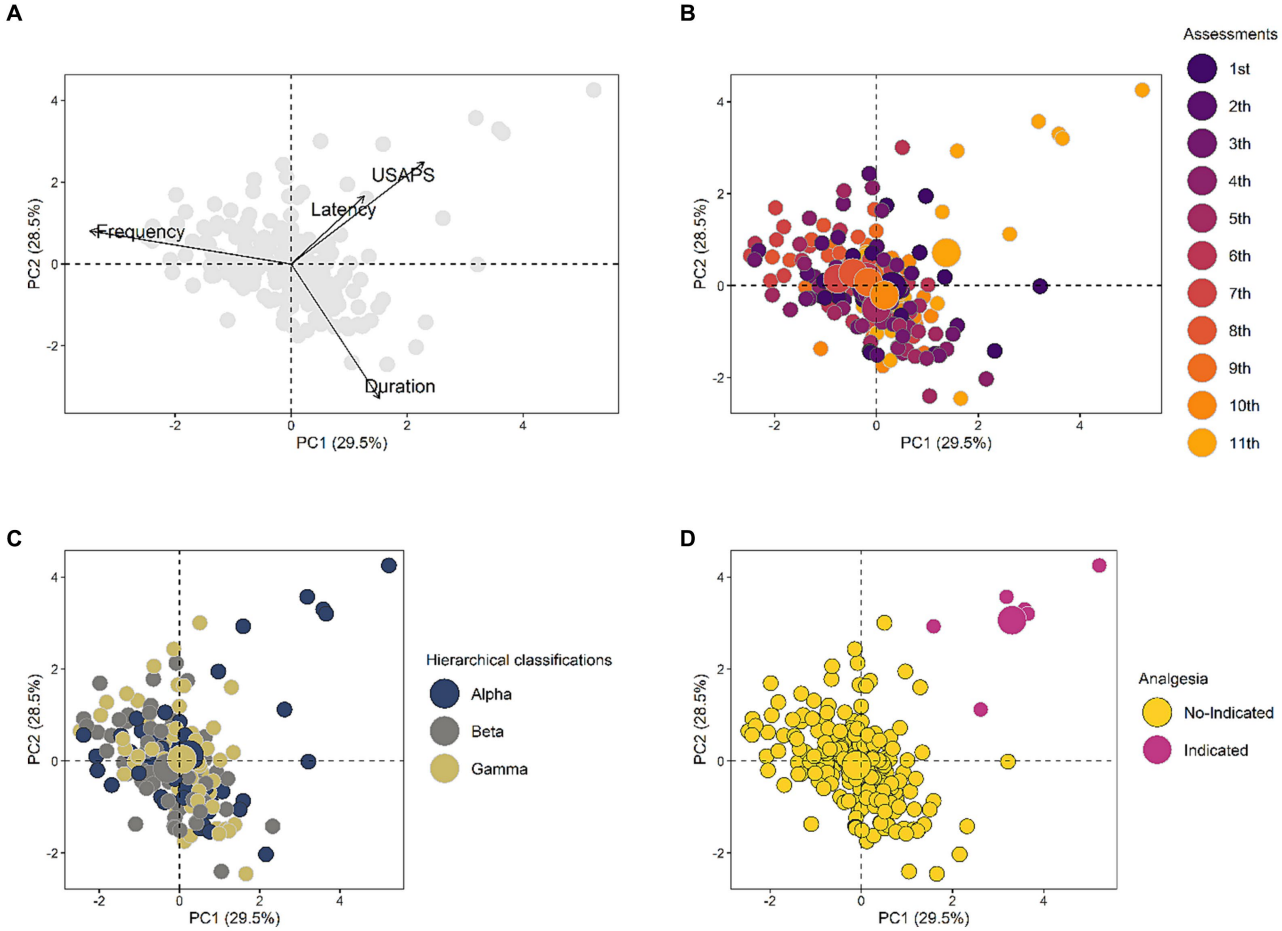
Two-dimensional scatter plot of principal component analysis of interrelationship of the variables **(A)**, distribution of rams segregated by evaluations **(B)**, hierarchical classification **(C)**, and analgesic indication **(D)**. The first through tenth evaluations represent the 2 batteries (5 d each) of behavioral tests conducted pre-orchiectomy, whereas the eleventh evaluation was post-orchiectomy. The smaller circles indicate each animal in each assessment, and the larger circles indicate the centroid. The centroid indicates the center of mass given by the polygon formed with the interpolation of the smaller circles of the same color. The arrows indicate the vectors of each variable.

Horn parallel analysis indicated 2 PC as optimal to be retained in the PCA. The first 2 PC (or dimensions) of PCA together captured 58% of the total variance, and in the PC1, there was a positive association between USAPS and duration. In contrast, duration was negatively associated with USAPS in the PC2 (Figure 4 and Table 1). In addition, the USAPS was located in the upper left quadrant, where there was a higher density (light orange larger circle) of the eleventh evaluation (post-orchiectomy), alpha rams, and with indication to receive analgesia (pink larger circle) based on a score higher than the USAPS cut-off point. The larger circle is the centroid, indicating the center of mass given by the polygon formed with the interpolation of the smaller circles of the same color. These results suggest that the UPAPS may have been positively related to postoperative pain.

**Table 1:** Loading values, eigenvalues, and variance of principal component analysis to explore multiple associations between variables used to measure interest in an extra feed resource in rams.

Parameters	PC1	PC2	PC3	PC4
Latency	0.30	0.39	0.87	0.02
Frequency	−0.82	0.19	0.14	0.52
Duration	0.36	−0.78	0.18	0.49
USAPS	0.54	0.59	−0.39	0.46
Eigenvalue	1.18	1.14	0.96	0.72
Variance	29.54	28.48	23.98	18.00
Accumulated variance	29.54	58.02	82.00	100.00

**Figure 5:**
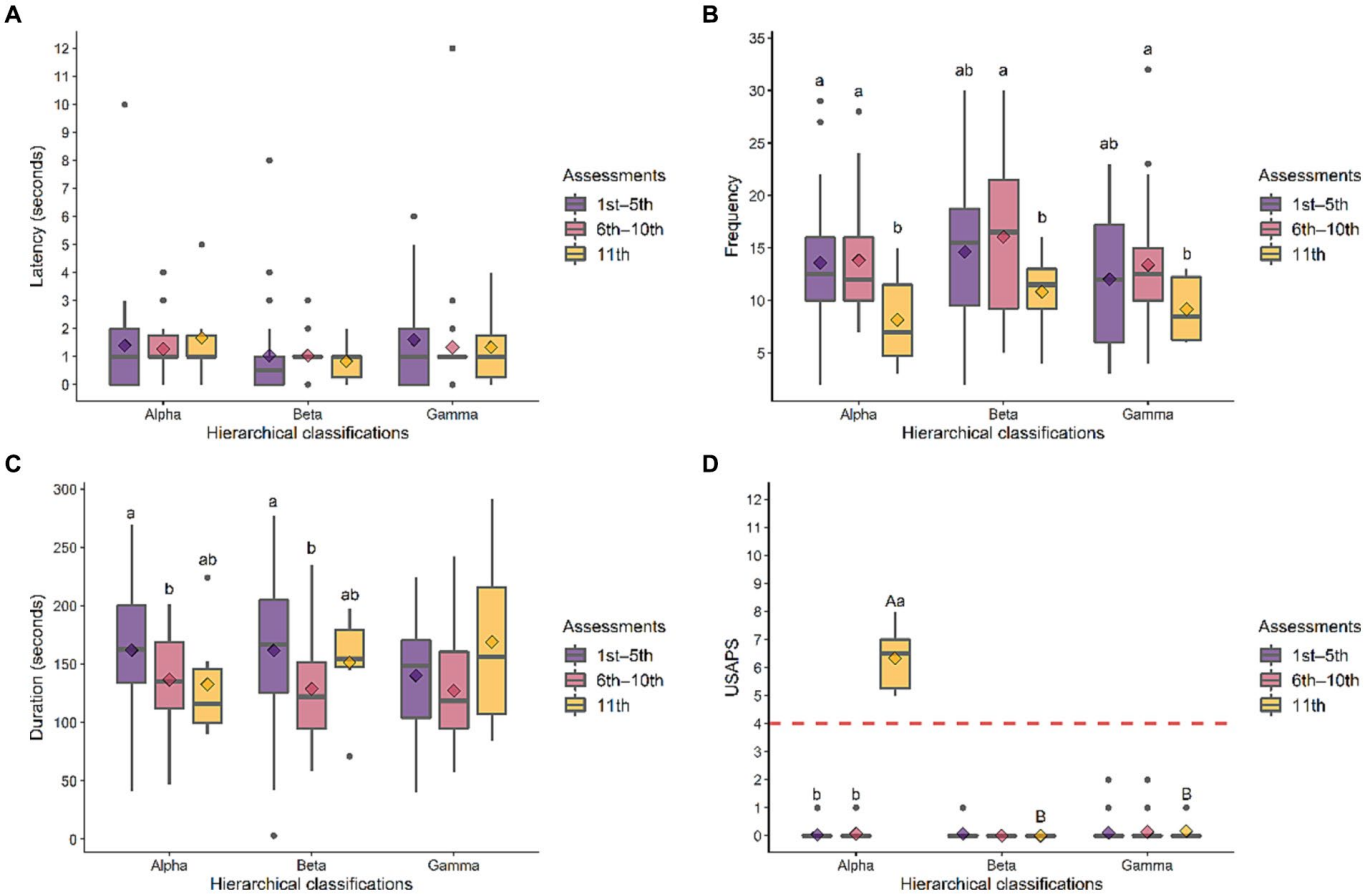
Box plot of latency **(A)**, frequency **(B)**, duration **(C)**, and Unesp-Botucatu sheep acute pain scale (USAPS) **(D)** over three assessment periods (1st–5th and 6th–10th preoperative, and 11th postoperative) in rams hierarchically classified as alpha, beta, or gamma. Lower and upper bounds of the box represent the first and third quartiles of the data, respectively; the longer horizontal line inside the box indicates the median; the diamond indicates the mean; gray circles indicate outliers; capital letters indicate differences between hierarchical groups in the same assessment (A > B); lowercase letters indicate differences between assessments in the same hierarchical group (a > b); multiple comparisons were carried out by the models with a post-test by Tukey’s test (*p* < 0.05); the red dashed line in plot D indicates the optimal USAPS cut-off for analgesia indication (≥ 4 points); only alpha rams were castrated, but all rams were evaluated in all assessments.

Latency was statistically equivalent over assessments and between hierarchical classifications (Figure 5A). Frequency decreased from the 6th–10th to the 11th assessment in all rams and was statistically equivalent between hierarchical classifications (Figure 5B). Duration decreased from the 1st–5th to 6th–10th assessments with no changes between hierarchical classifications (Figure 5C). USAPS was statistically equivalent across the 11 evaluations for beta and gamma rams, whereas for alpha rams, there was an increase in the final assessment. In the comparison between hierarchical classifications, the alpha had the highest pain score (Figure 5D), clear evidence that the rams experienced pain at 8 h post-orchiectomy. The distribution of variables over the 11 assessments can be seen in the Supplementary Figure S1.

## 4 Discussion

The novel aspect of this study was the investigation of the effect of postoperative pain on the interest of alpha rams in feed with limited access. Social interactions analyzed in the first and second testing batteries recognized levels of hierarchy among rams from the same group and consistently identified the alpha ram. After orchiectomy, alpha rams had high USAPS scores, confirming the expected pain, as predicted by the preoperative analgesic half-life (23). However, alpha rams do not have reduced interest in the highly contestable feed resource in the presence of acute postoperative pain, rejecting our hypothesis.

Behavioral agonistic interactions are used to determine social hierarchy in sheep (19, 25). The present study sought to identify pain in rams submitted to orchiectomy in a simpler, faster, and more practical way during the regular management of feed in animal husbandry. We encouraged a highly competitive environment, common on breeding farms, characterized by groups of confined males, with the offer of a preferable feed, with limited time and quantity, to stimulate the animals even in the presence of a painful stimulus, and analyzing its impact on access to the resource. Also, it should be noted that our rams were not subjected to fasting, as reported in some studies (21, 22, 26). As the rams classified as alpha had a higher social hierarchy index than those classified as beta or gamma, we concluded that our methodology effectively identified the dominant ram in each group.

The instrument adopted to diagnose acute pain (USAPS) (17) in the present study indicated pain exclusively for alpha rams at the evaluation 8 h after orchiectomy, predicted to be the peak of postoperative pain due to the duration of the preoperative analgesia effect (23). Although the USAPS was initially developed and validated to assess abdominal pain induced by laparoscopy in female sheep, we suggest that the instrument was sensitive and specific enough to diagnose pain due to orchiectomy in rams. Other behavioral pain assessment instruments that only consider facial expressions (27, 28) could not be applied during the behavioral test, as the rams had their heads down, chewing, with their muzzle and mouth inside the trough, making it impossible to analyze subtle facial changes. Therefore, due to the particularities of the experimental design, an instrument that considered whole-body language was used.

In the present study the frequency and duration that the alpha ram accessed the extra feed trough when diagnosed with pain were equivalent to the beta and gamma rams, suggesting that acute pain did not decreased alpha rams´ interest in a highly contestable feed. However, we observed a decrease of frequency in alpha rams in the 11th behavioral test, compared to the first and second previous assessment batteries, and in beta and gamma rams, compared to the second batterie. These findings suggest that other factors might influence these results (i.e., anhedonia - loss or reduction of the enjoyment of the reward). Pain can be a stress-inducing factor that modulates social interactions in laboratory animals (29). In pain, mice presented changes in social behavior and male dominance relationships (30, 31). Conversely, in the present study, the latency to touch the mouth on the extra feed trough presented low variation, based on analysis of principal components, suggesting that even with pain, the alpha ram took an equivalent time to access the feed trough compared to when in a pain-free state. Taking together, our findings signalize that acute pain did not decrease the interest of alpha rams in a highly preferable food. As this was apparently the first time that the effects of social hierarchy on pain assessment in sheep have been investigated, further studies are needed.

On commercial sheep farms, painful procedures (castration, tail docking, and mulesing) (3, 4, 10) are commonly conducted, and sheep are widely used in experimental studies involving pain conditions (2). They also experience pain due to unplanned occurrences (e.g., diseases, injuries, mastitis, hoof problems, and dystocia) that may go unnoticed (9). Therefore, the moment of feeding may be an excellent opportunity to detect pain-related behavioral changes that could be related to acute pain using the USAPS which combines different maintenance and specific pain or discomfort behaviors to quantify pain. However, this study highlighted that acute post-orchiectomy pain does not reduce alpha rams’ interest in feed resources, evidencing dominance might be a confounding factor in appetite-related behaviors used to diagnose acute pain assessment in alpha rams.

This study is not without some limitations. Although we evaluated only three sheep in each of the six groups, there were sufficient significant differences in diagnosing pain state. Despite this, future studies could make efforts to investigate group sizes more similar to those common in commercial herds. Additionally, in the present study, we analyzed the alpha ram because this is the most straightforward hierarchical position to identify and distinguish from beta and gamma rams, appearing to be an excellent way to start studying the confounding factor of hierarchy in ram pain assessment. We limited the post orchiectomy evaluation to just one section, because if we had evaluated alpha rams’ behavioral response more than 1 day after castration, this would probably had changed the social dynamics, based on the sharp decrease in testosterone 24 h post-castration reported in other mammals (32–34). In this case, the reduction in testosterone would represent a bias because endogenous testosterone levels modulate agonistic behaviors (35–37). Our study represents a first step in understanding the confounding factor of social hierarchy on pain assessment in sheep, which has never been previously studied. The whole-body behavior, facial expressions, and physiological responses (e.g.: testosterone, serum biomarkers of inflammation and heart rate) of the alpha, beta, and gamma rams should be considered in future studies.

## 5 Conclusion

We concluded that alpha rams’ interest in accessing a limited feed resource has not decreased as an effect of the acute post-orchiectomy pain. During routine husbandry under field conditions, feeding could be an excellent opportunity to detect pain-related behavioral changes based on the USAPS, as rams exhibit behaviors associated with acute pain even during access to limited, highly preferable, and contestable feed resources. However, our findings suggest dominance might be a confounding factor in appetite-related behaviors detecting acute pain assessment in alpha rams, as the acute pain did not affect their interest in accessing limited feed resources.

## Data availability statement

The raw data supporting the conclusions of this article will be made available by the authors, without undue reservation.

## Ethics statement

The animal study was approved by the Ethics Committee on the Use of Animals - FMVZ - Unesp. The study was conducted in accordance with the local legislation and institutional requirements.

## Author contributions

KU: Data curation, Investigation, Visualization, Writing – original draft. PT: Conceptualization, Data curation, Formal analysis, Funding acquisition, Investigation, Methodology, Project administration, Writing – original draft, Writing – review & editing. PR: Investigation, Project administration, Writing – original draft, Writing – review & editing. AC: Data curation, Investigation, Writing – original draft. LB: Investigation, Writing – original draft. MT: Investigation, Writing – original draft. HG: Investigation, Writing – original draft. AP: Investigation, Project administration, Writing – original draft. RD: Investigation, Writing – original draft. ER: Investigation, Writing – original draft. JK: Funding acquisition, Writing – review & editing. JF: Conceptualization, Funding acquisition, Methodology, Supervision, Writing – original draft, Writing – review & editing.

## Footnotes

^1^https://animalpain.org/en/ovelhas-dor-en/

^2^https://play.google.com/store/apps/details?id=com.vetpain.app
