# Supplementary material for: Acute post-orchiectomy pain does not reduce alpha rams’ interest in feed resources
Source: Front Vet Sci. 2024 Mar 19;11:1299550. doi: 10.3389/fvets.2024.1299550 (PMC10985335; doi:10.3389/fvets.2024.1299550)
Supplement: Supplementary file 1 [file Data_Sheet_1.pdf]

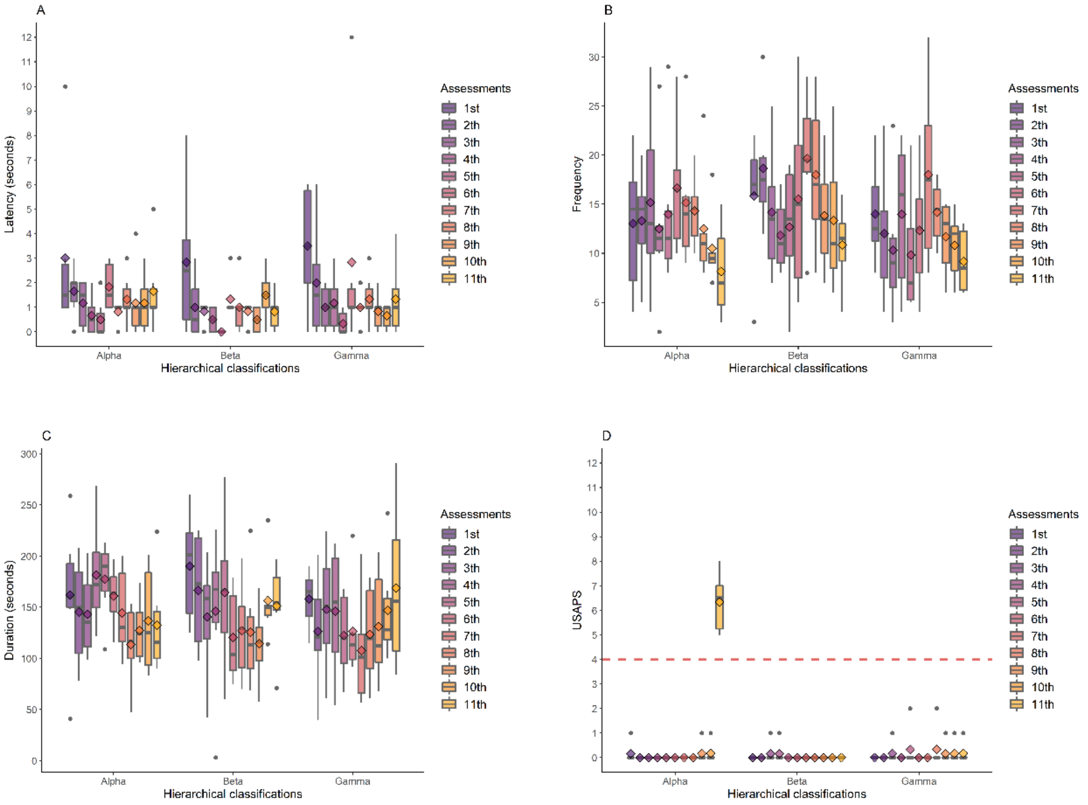

Figure S1 - Box plot of latency (A), frequency (B), duration (C), and Unesp-Botucatu sheep acute pain scale (USAPS) (D) over 11 assessments (1st–10th preoperative, and 11th postoperative) in rams hierarchically classified as alpha, beta, or gamma. Lower and upper bounds of the box represent the first and third quartiles of the data, respectively; the longer horizontal line inside the box indicates the median; the diamond indicates the mean; gray circles indicate outliers; the red dashed line in plot D indicates the optimal USAPS cut-off for analgesia indication (≥ 4 points); only alpha rams were castrated, but all rams were evaluated in all assessments.
